# The Impact of the COVID-19 Pandemic on Ophthalmology Residents: A Narrative Review

**DOI:** 10.3390/ijerph182111567

**Published:** 2021-11-03

**Authors:** Natalia Dub, Joanna Konopińska, Iwona Obuchowska, Łukasz Lisowski, Diana Anna Dmuchowska, Marek Rękas

**Affiliations:** 1 Department of Ophthalmology, Medical University of Bialystok, 15-276 Bialystok, Poland; natalkand@wp.pl (N.D.); iwonaobu@wp.pl (I.O.); lisowski@vp.pl (Ł.L.); diana.dmuchowska@umb.edu.pl (D.A.D.); 2 Department of Ophthalmology, Military Institute of Medicine, 04-141 Warsaw, Poland; rekaspl@gmail.com

**Keywords:** COVID-19, ophthalmology residency program, mental health, ophthalmology trainee, virtual training

## Abstract

The ongoing outbreak of the coronavirus disease 2019 (COVID-19) pandemic has drastically affected medical societies. We aim to provide an overview and summarize the information published so far concerning the impact of the COVID-19 pandemic on ophthalmology residency programs and the mental wellbeing of trainees, and to establish factors to help maintain successful residency training to ensure high-quality, specialist ophthalmic training. A literature search was conducted in October 2021 of the PubMed database for articles assessing the impact of the COVID-19 pandemic on the mental health of ophthalmology trainees and on ophthalmology residency programs. Cross-sectional survey studies, editorials, articles in scientific journals, letters to editors, and commentaries were considered; finally, 19 studies were included after excluding abstract-only publications and conference posters. The studies’ demographic details, participant characteristics, interventions, outcomes, and limitations were extracted. Our summarized information showed the alarmingly significant impact of the COVID-19 pandemic on ophthalmology trainees’ mental health and the associated considerable changes in ophthalmic training programs. Thus, in future, virtual training and surgical simulators should be permanently introduced, in addition to traditional teaching, to complete successful ophthalmology residency programs. Additionally, we emphasize the need for a widely facilitated and encouraged access to psychological support programs for healthcare workers, including ophthalmologists.

## 1. Introduction

The novel coronavirus, severe acute respiratory syndrome coronavirus 2 (SARS-CoV-2), responsible for coronavirus disease 2019 (COVID-19), was first identified in China at the end of 2019 [1]. The rapid spread of COVID-19, resulting in a global threat to public health, led the World Health Organization to declare the disease a pandemic on 11 March 2020 [2]. The growing number of people affected with SARS-CoV-2 worldwide prompted immediate action to divert healthcare resources towards the treatment of patients with COVID-19 and to limit the spread of the disease.

Over the first few months of the pandemic outbreak, regarding ophthalmology societies, most ophthalmic departments suspended their elective clinical and surgical activities and restricted their practices to urgent cases only [3,4,5]. Moreover, the emergency situation in many places led to rotations of healthcare personnel, including trainees, who were redeployed to COVID-19 wards to fight the pandemic.

The worldwide crisis within healthcare systems had a knock-on effect on all aspects of graduate medical education, including residency programs, especially for practical activities and the didactic teaching of trainees [6,7,8]. Despite the slow reopening of routine eyecare services, based on local scenarios and public health guidelines, over the following few months of the ongoing pandemic, the restrictions established in public areas and social distancing rules were still in force. This led to the introduction of different methods of virtual teaching, which included, among others, the organization of courses, conferences, and lectures through online platforms.

**Figure 1:**
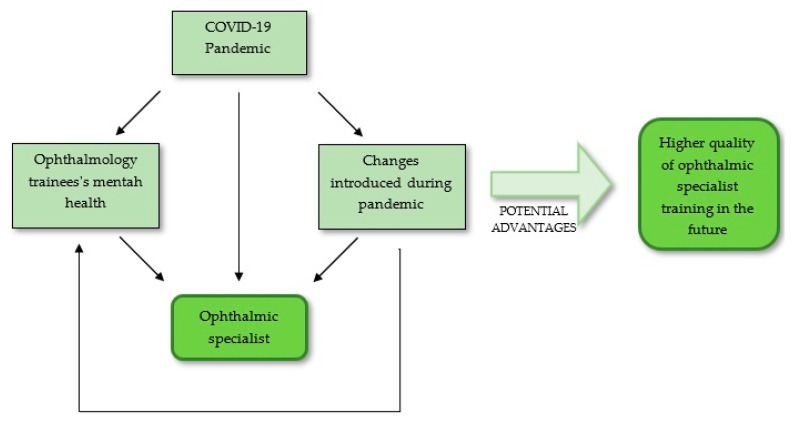
Diagram presenting the main idea of our study.

Several studies from different countries showed the impact of COVID-19 on ophthalmology training programs and trainees’ mental health, mostly focusing on the perspectives of the trainees [6,7,8,9,10,11,12,13,14,15,16]. Although significant, the problem is relatively new. Considering the above, we aim to provide a short overview of the literature concerning trainees’ opinions and experiences with the changes in ophthalmology training and their mental wellbeing in relation to the current pandemic (Figure 1). To the best of our knowledge, this is the first study providing an overview of the current literature assessing the impact of the COVID-19 pandemic on ophthalmology specialist training, based mainly on survey studies published thus far.

**Figure 2:**
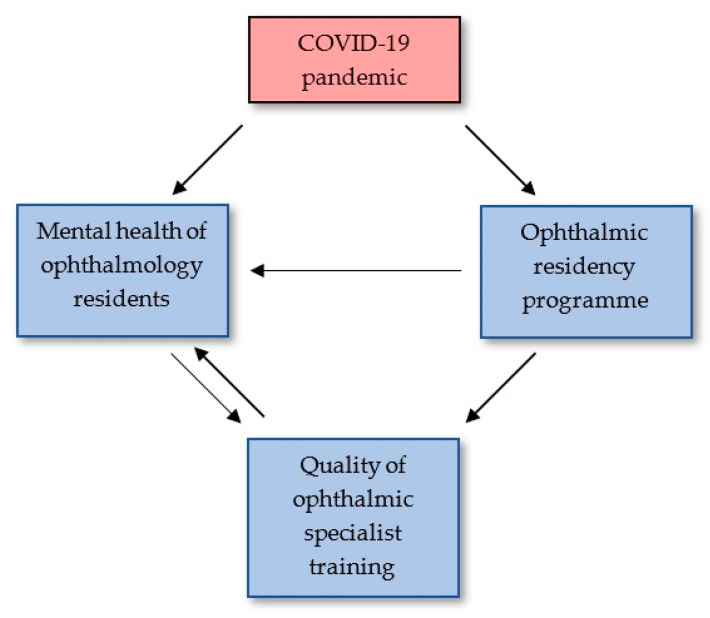
Diagram illustrating the impact of the COVID-19 pandemic and the interaction of the mental health status and ophthalmology residency programs.

The COVID-19 pandemic impacts the quality of training not only by affecting the programs but also by indirectly affecting the mental health of both teachers and trainees. This in turn affects the quality of teaching and training. Furthermore, this reduced quality may affect mental health, possibly leading to a vicious circle (Figure 2). As the factors of both mental health and training programs interact, they require special attention. We believe that the summarized information will potentially indicate beneficial, long-term solutions that could be introduced permanently in the future to maintain successful residency programs. 

## 2. Materials and Methods

### 2.1. Literature Search

We searched PubMed, Web of Science, Cochrane Library, and ClinicalTrials.gov to identify relevant studies, on 15 October 2021. The following terms were searched in each database: “ophthalmology residents,” “ophthalmology trainee” or “ophthalmologists mental health” or “ophthalmology training program,” and “COVID-19” or “SARS-CoV-2.” We did not apply restrictions for language or date of publication. 

### 2.2. Study Selection

Articles that assessed mental health issues faced by ophthalmology trainees during the current pandemic and the pandemic’s impact on ophthalmology residency programs were included in this review. Due to the relatively short study period (since the outbreak of the pandemic), the publication types that we considered included cross-sectional survey studies, editorials, articles in scientific journals, letters to editors, and commentaries. The abstracts of the found articles were assessed for relevance, and references were screened to identify articles that needed manual search. Publications that were only available as abstracts or conference posters were excluded. Consequently, 19 full-text, original articles and those related to the topic were selected. None of the relevant articles were excluded based on language. Full-text translation was performed, if necessary. 

**Figure 3:**
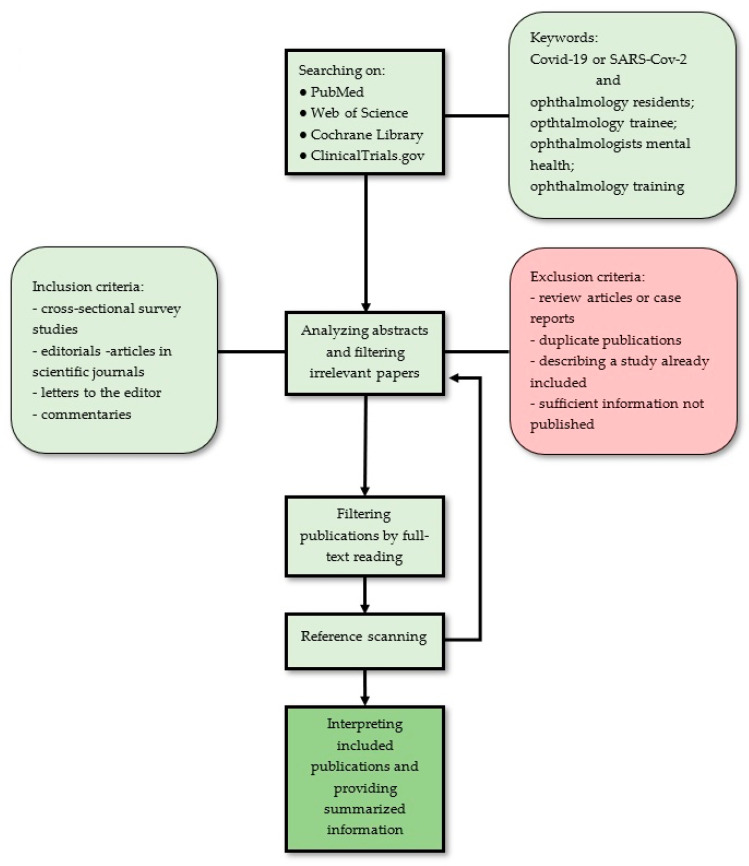
Flow graph illustrating proposed approach.

**Figure 4:**
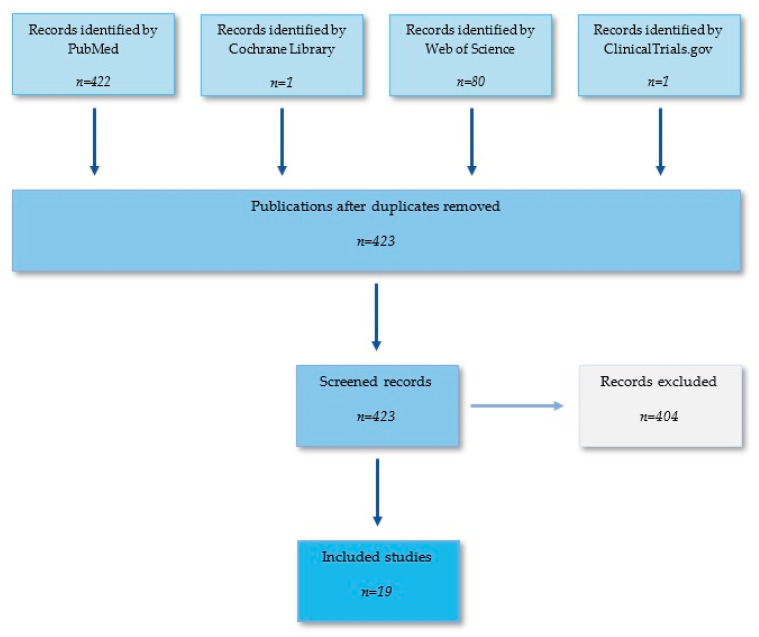
Flow graph illustrating searching method.

Exclusion criteria were as follows: (1) review article or case report, (2) duplicate publications, (3) describing a study already included (e.g., subgroup analysis or mid-term report of a large trial, or follow-up report after a trial ends), and (4) sufficient information not published (e.g., full text not accessible or full text did not contain raw data), (Figure 3 and Figure 4). 

### 2.3. Risk of Bias Assessment

The methodological quality of the included studies was independently evaluated by two authors (ND, JK).

### 2.4. Data Extraction

The studies’ demographic details, participant characteristics, interventions, outcomes, and limitations were independently extracted by two authors (ND, JK). Disagreements, if any, were discussed and resolved through discussion.

## 3. Results

### 3.1. Impact on Ophthalmology Trainees’ Mental Health

There are few studies in the literature assessing the impact of COVID-19 on mental health and ophthalmology residency programs from the resident’s perspective by using online questionnaires. Most studies have brought considerable attention to the negative psychological consequences of the current pandemic (Table 1).

We found three survey studies assessing psychological distress among ophthalmology trainees using the Patient Health Questionnaire-9, which is considered to be a valid and reliable tool to assess depressive symptoms [9,10,11,12]. Alarmingly, in a study conducted by Alahmadi et al. [9], nearly half of the participants reported mild-to-moderate depressive symptoms. Khanna et al. [10] found that many survey responders (approximately 32%), including more than 2000 ophthalmologists and ophthalmology trainees who participated in their study, were psychologically affected. Almater et al. revealed a significantly higher occurrence of depression and elevated levels of stress, anxiety, and insomnia [11]. 

Accordingly, Mishra et al. [13] reported that almost 55% of their study participants indicated higher stress levels, and approximately 47% of the responders felt ‘unhappy’ during the lockdown.

A study conducted by Szigiato et al. [14] showed that most trainees had higher levels of anxiety during the pandemic compared with levels before the pandemic (70% agree/strongly agree). Similarly, high anxiety levels were detected in Turkish ophthalmologists in a cross-sectional study conducted by Kavadarli et al. [15]. Similarly, most of the responders from Cairo hospitals had a higher level of anxiety related to the pandemic after grading their psychological concerns [16]. The above-mentioned study, conducted among young ophthalmologists, revealed that almost 56% felt unlucky starting ophthalmic practice during the COVID-19 era, whereas 19% regretted becoming physicians, and 30% even considered a career shift [17].

Interestingly, one survey study conducted by Hussain et al. [18] among trainees in the United Kingdom revealed that most participants insisted that COVID-19’s impact on their ophthalmic training had no effect, or even a positive effect, on their mental health (46% and 8%, respectively), whereas 46% agreed that the current pandemic had a negative impact on their wellbeing. Compared to these results, only 5.6% of trainees from Saudi Arabia stated that COVID-19 had no impact on their mental health [9].

A few of the reviewed studies tried to discover the factors that may cause a significant influence on psychological aspects. El-Saied et al. [16] found that factors such as the respondents’ age, duration of ophthalmology practice, and sex had a statistically significant impact on many aspects such as psychological concerns, psychological assessment, and their desire to volunteer in the intensive care unit (ICU); a feeling that they needed a psychological assessment was commonly observed in young ophthalmologists, especially those with 1–3 years of practice and among females. A willingness to volunteer in the event of medical staff shortages was more frequent in those with 6–9 years of experience in ophthalmic practice, particularly among males. Another study by Khanna et al. [10] also showed the impact of the respondent’s age on the onset of depression related to the COVID-19 pandemic, which was significantly higher in younger ophthalmologists, and the odds of depression decreased by 3% with a 1-year increase in age. A survey study conducted by Almater et al. [11] revealed that working in a frontline healthcare was significantly connected with the occurrence of insomnia and anxiety; the factor of female sex was strongly connected with the occurrence of depression, stress, and anxiety. Furthermore, living with elderly people was associated with a higher occurrence of depression and anxiety.

Examples of the COVID-19-related, specific challenges to trainees and the approaches to solving them were described. Lim et al. [19] revealed that the redeployment of ophthalmologists to other areas of need during the COVID-19 pandemic was associated with increased anxiety levels, especially if special training was not performed. For comparison, up to 40.5% of responders from Cairo hospitals declared themselves willing to volunteer in the case of medical staff shortages to help in emergency departments, ICUs, or hospital wards [16]. Interestingly, trainees from the University of Iowa in the United States, who first feared being moved to internal medicine floors or ICUs, described that, later, despite their fear lessening, they experienced feelings of guilt after the decision of that ophthalmology trainees would not be called to manage inpatients in the United States [20]. They stated that they felt ashamed because they suddenly had time to care for themselves, while several individuals around them were suffering. Other concerns described by Szigiato et al. [14] among trainees included the fear of spreading the virus to patients (74% agree/strongly agree) or family and friends (86% agree/strongly agree). Moreover, the feelings of isolation and loss of the integral community due to the introduced restrictions during lockdowns were also observed [13,17]. Rakowsky et al. [21] described the chief trainees who emphasized their role in helping trainees remain connected and unified regardless of their clinical rotation, which was conducted by encouraging teams in the hospital and implementing online discussion forums and video conferencing to achieve these goals. Most of the studies agreed that training institutes and ophthalmology societies should be aware of the need to support mental health and must offer psychological counselling and psychiatric support to all healthcare workers, including ophthalmology trainees.

### 3.2. Impact on Residency Training Programs

Due to the COVID-19 pandemic, a certain number of ophthalmology trainees were redeployed to emergency departments or ICUs, temporarily interrupting their ophthalmic training (Table 2). For those who were not redeployed, they were frequently organized into small team units working separately and communicating via phone or online platforms, reducing cross-exposure risks [14]. In some cases, teleophthalmology was introduced for screenings and basic visits [22,23,24].

A survey study conducted by Nair et al. [23] in India during a nationwide lockdown, including more than one thousand ophthalmologists, showed that almost 73% of the respondents (913/1260) did not even see any patients during the lockdown. More than half of the young ophthalmologists in Cairo Hospital reported that the COVID-19 pandemic decreased education and training programs by 50–75% [16], which was in line with the the results of other recent studies where participants believed that their training was severely affected [7,10,15].

According to recently published data from ophthalmology residency programs worldwide, a significant reduction in routine outpatient care and a decrease in surgical exposure and cancelation, or a significant reduction in elective surgeries, which were limited only to urgent or emergency cases, was reported [4,7,10,12,15,19,20]. Approximately 80% of the respondents in a survey study conducted in India confirmed that the lockdown had a negative impact on their surgical training, and almost half of the surveyed trainees in Portugal did not assist in any surgical procedures, excluding intravitreal injections [23,25]. Our previous study conducted among residents in Poland also revealed that the majority (88.9%) of them felt that the COVID-19 pandemic had negatively impacted their surgical training with total or partial restrictions of elective surgeries reported by 92.8% of them [26]. A recent survey among ophthalmology trainees in the United Kingdom to assess the impact of COVID-19 on training showed that the lack of cataract surgery training was the single most frequently raised concern [18,19].

In a cross-sectional survey conducted by Szigiato et al. [14], most Canadian ophthalmology trainees (>50%) stated that they did not have the adequate availability for surgical simulation or access to a wet laboratory to maintain their surgical skills. In contrast, trainees from the United States described taking advantage of the clinic restrictions as they began investing more time in surgical simulation training, proactively scheduled by their faculty and individualized to each resident’s level of training. They stated that, despite having limited surgical experience during the coronavirus pandemic, a quick transition to a simulation curriculum allowed the maintenance of progress in surgical skills and continued the resident–faculty interaction [20].

#### 3.2.1. Research Work

Silva et al. [25] reported that most of the respondents (80%) declared having more time to develop research projects during the pandemic, and an increase in research productivity was found in 40% of the trainees. In contrast, in our previous study [26] 88.9% of surveyed residents, who were involved in research activities before the pandemic, confirmed the negative impact of the pandemic on their research work.

#### 3.2.2. Online Teaching Methods

In a survey study conducted in India [13], trainees were instructed to quantify the impact of the pandemic on their training program: the effect on classroom/theoretical learning was assessed as less than 50%, in contrast to their surgical training, which most trainees (62.4%) felt had decreased by 50% or more. Approximately 75% of respondents found ophthalmology webinars and online classes conducted during this lockdown period to be useful [27]. Similarly, Saudi Arabian ophthalmology trainee feedback on web-based training during the COVID-19 pandemic suggested that more than half of the trainees were satisfied or highly satisfied [11]. Another cross-sectional study conducted in India, assessing the impact of the webinars on the residents’ learning during the pandemic, revealed that 75% of residents positively graded webinars as a good/very good academic tool and nearly 77% of them admitted that they gained substantial knowledge of the topic in question [28]. The positive perception and usefulness of webinars in enhancing theoretical knowledge and practical skills were also confirmed in another survey study among Indian ophthalmology residents conducted by Mishra et al. [13,17]. They revealed that 74% of residents preferred webinars based on their residency curriculum with clinical problem-solving as the specific area of interest (40.9%) and the ideal duration of webinars between 60 and 120 min [13]. Similarly, regarding the usefulness of the abovementioned online teaching method, a cross-sectional study conducted by Rana et al. [27] revealed that most (72.8%) of surveyed ophthalmologists reported a knowledge gain from webinars, with nearly half of the respondents assessing them as good to excellent, with retina- and cataract-themed webinars as the most beneficial. However, most of the respondents stated that there was a repetition of the same topics in the webinars and admitted to becoming confused regarding “which webinars to attend and which not to”.

#### 3.2.3. Perspectives

Mishra et al. [13] suggested that permanent changes, such as virtual classrooms and simulation-based training, should be considered. Similarly, in a study conducted by Hussain et al., most of the surveyed trainees stated that the academic lectures and seminars could be replaced by online sessions after the COVID-19 pandemic [18]. Considering the form of online teaching methods, trainees from Cairo preferred recorded lectures that could be retrieved at any suitable time (63%) compared to live, online, interactive lectures (37%) [16]. Similarly, in a study conducted by Dasgupta et al. [28], more than half of the surveyed residents (54%) preferred to attend webinars after the COVID-19 pandemic and nearly 43% of them felt that webinars should run parallel with face-to-face classroom teaching. In a study conducted by Rana et al. [27], the majority of respondents felt that webinars should continue after the COVID-19 pandemic. However, the authors emphasize the need for improvisation in the current pattern of webinars considering inter alia the linking of webinars to ophthalmology boards, a better presenter–attendee interaction, and the novelty of content. The positive perception and usefulness in enhancing the theoretical knowledge and practical skills from webinars was also confirmed in another survey study among Indian ophthalmology residents conducted by Mishra et al. [14] They revealed that 74% of residents preferred webinars based on their residency curriculum with clinical problem-solving as a specific area of interest (40.9%) and an ideal duration of webinars of between 60 and 120 min [14]. A study conducted among trainees in Portugal showed the residents’ perspectives regarding strategies that could be performed to minimize the negative impact of the pandemic. Most respondents agreed with the extension of the training program (80%), a curriculum incorporating targeted surgical procedures (56%), and the development of virtual platforms for educational courses (52%), as well as future simulation training programs (44%), virtual platforms for congresses and webinars (41%), and virtual platforms with educational surgical videos (39%), which were also significantly selected [25].

The suggestions from trainees surveyed in the United Kingdom on how ophthalmic specialist training could be improved during the pandemic included, among others, more simulations and wet laboratory opportunities [17,18]. Similarly, trainees from Cairo mostly preferred simulation training compared to wet laboratories and video sessions [16].

Considering the changes in the research related to the pandemic, Kumar et al. [29] suggested that institutions could formulate revised guidelines for all patient-based clinical studies to make their sample size feasible, which would help trainees to complete their projects.

## 4. Discussion

The summarized information in our article shows the alarmingly significant impact of the COVID-19 pandemic on ophthalmology trainees’ mental health and the associated, considerable changes in ophthalmic training programs.

A substantial occurrence of depressive symptoms, increased anxiety, and negative thoughts among young ophthalmologists was widely detected in the reviewed studies. It is well known from studies conducted in the past concerning the mental wellbeing of healthcare workers related to the pandemic, and reviews involving healthcare workers during the COVID-19 pandemic, that healthcare workers are at a higher risk of adverse psychiatric implications, especially those working in emergency units, ICUs, and infectious disease wards [30,31]. Liang et al. [32] showed that there was no significant difference in the occurrence of anxiety and depression among medical staff in the COVID-19-associated units and other departments, which was consistent with the frequent presence of these symptoms in ophthalmology trainees.

Similar to the findings in the surveyed ophthalmology trainees, other studies conducted among healthcare workers also showed a positive association between female sex, younger age, higher self-rated depression scores, anxiety, and distress [33,34]. A cross-sectional study, conducted by Wan et al. in China among healthcare trainees, revealed a positive association between active clinical duties during the pandemic and feelings of distress, particularly among medical trainees and undergraduates [24]. In contrast, the appearance of depressive feelings was found to be higher in non-practicing ophthalmologists, especially those who were considerably worried about their training or professional growth [10].

The supply of adequate personal protective equipment (PPE) and implementation of protocols may enhance the wellbeing of healthcare workers [34,35,36,37]. Wang et al. [35] also found that up-to-date and accurate health information (e.g., treatment, local outbreak situation) and precautionary measures (e.g., hand hygiene, wearing a mask) were associated with a lower psychological impact and lower stress, anxiety, and depression in the general Chinese population. Consistent with these findings, a study conducted by Mishra et al. [17], wherein stress levels among participants were significant, showed that approximately 65% of those who were on COVID-19 duties felt that the PPE they were using was inadequate. In contrast, most surveyed trainees in Saudi Arabia experienced depressive symptoms, which were noted in over half of them, but were provided with adequate PPE, built-in shields for slit lamps, and updated protocols on how to limit SARS-CoV-2 transmission [9,11]. Similarly, most trainees (93.0%) in Canada, where high levels of COVID-19-related anxiety existed, felt that they had adequate access to PPE [14]. In line with these findings, more than half of the Turkish ophthalmologists surveyed in a study conducted by Kavadarli et al. [15], which exposed the high levels of anxiety regarding the pandemic, stated that PPE was generally sufficient (53.7%) but 79% of them were not trained in the efficient use of PPE. A study conducted in France by Vallée et al. [35], where the impact of COVID-19 on mental health and the training of young surgeons was assessed, revealed that sufficient PPE and training regarding COVID-19 were statistically associated with decreased mental distress. Considering the accurate information transmitted to healthcare workers, a previous study revealed that the vast majority of ophthalmology trainees trusted infectious disease specialists, virologists, and immunologists regarding the COVID-19 pandemic [26,38]. However, the sources from which the trainees derived their knowledge regarding SARS-CoV-2 were not from the abovementioned specialists and included the Internet (63% of responders), television, and radio broadcasts (13.5%), as well as family and friends (19.8%). Additionally, Rakowsky et al. [21] emphasized the role of the chief trainees in reducing inconsistencies in messaging, as the overload of information coming from both within and outside the hospital increased fear and doubt.

Considering the changes in ophthalmic residency training caused by COVID-19, it is necessary to mention that the relative decline in outpatient visits among all specialties was found to be the largest in ophthalmology, reaching 79% [39]. Because ophthalmologists were considered to be at a high risk of COVID-19 infection due to direct contact with patients during examination, many international and national ophthalmic organizations had established recommendations concerning patient management at the beginning of the pandemic [36,40]. These regulations resulted in a significant limitation of ophthalmic examinations in urgent cases. The need for social distancing and the minimization of in-person evaluations led to telemedicine becoming the primary and safest form of outpatient clinical care in healthcare settings. Hence, in some cases, teleophthalmology, which was previously reserved used for rural or underserved areas, was employed for screening and basic visits and became an opportunity for trainees to gain experience with telemedicine, which undoubtedly played an increasingly essential role in their careers [17,18,19,31,32].

Due to the almost complete suspension of elective surgeries and a significant reduction in emergency surgeries, the role of simulators in surgical training significantly increased [41,42,43,44,45]. It is well known that simulator training positively impacts the resident’s surgical outcomes, and trainees with simulation exposure are reported to have significantly lower complication rates [42]. This type of surgical teaching is well-received among trainees, as are different types of theoretical online teaching. Its usefulness was confirmed to increase considerably with Zoom (the most commonly used platform) [43], which is in line with reviewed studies showing the regular participation of trainees in virtual meetings.

A study on virtual education by Chatziralli et al. [45] suggested that because ophthalmology a surgical specialty, it requires direct teaching on the operating ward in the operating theatre, which cannot be completely replaced by virtual methods. Nevertheless, the positive reception of new learning methods shows that it should be permanently introduced after the pandemic on some level, in addition to traditional teaching to maintain a successful residency program.

Our study has some limitations. First, some of the reviewed studies assessing mental health did not investigate whether the described depressive symptoms were new or due to pre-existing conditions. Second, a few survey studies were conducted among young ophthalmologists, including residents and physicians, after specialist training (not solely residents). Moreover, survey studies were conducted during various periods of the pandemic and the types of restrictions in respective countries (full lockdown or lifting restrictions), and the course of the pandemic in particular regions, varied. These factors could affect the differences in the trainees’ opinions.

The impact of the pandemic on education among ophthalmologists may raise the question of whether trainees who complete the 5-year specialization period are sufficiently prepared to work with patients. Until the end of the pandemic, efforts should be made to ensure that the education of trainees is as effective as possible despite pandemic-related limitations, in order to prevent a potential negative impact on the care provided to ophthalmology patients. 

Based on our conclusions [26] we formulated the following recommendations to maintain the quality of ophthalmic specialist training during the pandemic:

1. Intensifying surgical skills training from the first year of training;
2. Introducing virtual surgical simulators (i.e., cataract surgery simulators) to all ophthalmology departments to develop practical skills among trainees;
3. Providing additional surgical training for trainees redeployed to COVID-19 units during the pandemic;
4. Introducing and improving virtual forms of teaching on a large-scale, including wet labs, virtual simulators, webinars, podcasts, vodcasts, online courses, and symposia.

In summary, most trainees in the reviewed studies expressed positive opinions regarding online learning utilities in clinical practice and a willingness to use online teaching methods after the COVID-19 pandemic. Therefore, in the future, virtual training and surgical simulators should be permanently introduced, in addition to traditional teaching, in order to complete ophthalmology residency programs.

All healthcare workers, including ophthalmology residents, who were not in the frontline of the COVID-19 pandemic, suffered mental health consequences due to multiple, and often individual, factors. In line with the reviewed studies showing a significant proportion of ophthalmology residents affected by the COVID-19 pandemic, we underscored the need for widely facilitated and encouraged access to psychological support programs.

Changes to ophthalmology residents induced by the COVID-19 pandemic open new perspective for research. Considering the limited literature on the effect of the COVID-19 pandemic on ophthalmology residency training thus far, and the useful conclusions derived from ongoing studies, there is a need to conduct more original studies in the future assessing the above issue to find and evaluate useful solutions. Identification of the most beneficial programs for trainees’ online teaching and establishing precise recommendations could ensure a higher quality of ophthalmology specialist training.

## Figures and Tables

**Table 1:** Survey studies showing negative impact of COVID-19 pandemic on ophthalmology trainees’ mental health.

Reference	Participants	Respondents	Psychiatric Symptoms	Main Findings
El-Saied et al., 2020 [16]	79	Trainees and young ophthalmologists (practicing for <1 to 9 years)	Negative feelings	There were 55.8% who felt unlucky starting ophthalmic practice during the COVID era; 11.4% regret being ophthalmologists; 19% regret being physicians; 30.4% think about a career shift; 21.8% think they might need a psychological assessment and help after the pandemic.
Alahmadi et al., 2020 [9]	142	Residents	Depression	Mild depression: 33.1%; moderate depression: 26.1%; severe depression: 11.3%.
Khanna et al., 2020 [10]	2355	Ophthalmologists, ophthalmology trainees; 358 (15.2%) were residents or fellows under training	Depression	Mild depression: 21.4%; moderate depression: 6.9%; severe depression: 4.3%. Depression significantly higher in younger individuals, females, those not in practice, ophthalmologists-in-training, and those who were single.
Szigiato et al., 2020 [14]	102	Trainee	Anxiety	Higher level of anxiety than before pandemic: 70%;
Mishra et al., 2020 [13]	716	Trainee	Stress	increased stress levels: 54.8%; generally unhappy state of mind during lockdown period: 46.5%.
Kavadarli et al., 2021 [15]	161	Actively working ophthalmologists with 28.4% ophthalmologists at the age of 25–34 years	Anxiety	Ninety-one percent of the participants stated that their anxiety level increased during the pandemic, most commonly due to the risk of transmitting the disease to their family (83%).
Almater et al., 2020 [11]	118	Ophthalmologists with varying practical experience, and the majority are residents (61.7%)	Depression Anxiety Insomnia Stress	Depression in 50.5% of respondents (21.4% reported severe and moderately severe symptoms; 17.8% with moderate symptoms and 21.5% with mild symptoms). Depression significantly higher among females, participants living with the elderly. Anxiety in 46.7% of participants (25.2% with mild symptoms, 15.9% with moderate, and 5.6% with severe symptoms). Anxiety significantly more common among females, frontline healthcare providers, and those living with elderly. Insomnia in 44.9% of respondents (29.9 with mild symptoms, 13.1% with moderate and 1.9/5 with severe symptoms), more commonly in front-line healthcare providers. Higher level of stress in 22% of respondents (28% with low level, 68% moderate level and 3.7% with severe); more common in females.

**Table 2:** Percent of ophthalmology trainees redeployed to areas of need.

Reference	Country/Region	Redeployment (%)
Silva et al., 2020 [25]	Portugal	25%
Szigiato et al., 2020 [14]	Canada	4.9%
Mishra et al., 2020 [13]	India	24.6%
Ferrara et al., 2020 [6]	32 different countries	27–28%
Alahmadi et al., 2020 [9]	Saudi Arabia	18.3%
Hussain et al., 2020 [18]	United Kingdom	39%

## Data Availability

Readers can access the data supporting the conclusions of the study upon an e-mail request to the corresponding author.
